# The Report of Engagement in Community Research (REACH) Tool: A Strategy to Promote Transparency and Improve Planning in Community-Engaged Research

**DOI:** 10.35844/001c.140734

**Published:** 2025-08-21

**Authors:** Farrah Jacquez, Lisa Vaughn

**Affiliations:** 1 Psychology, University of Cincinnati; 2 Emergency Medicine, Cincinnati Children’s Hospital & Medical Center, University of Cincinnati; 3 Educational and Community-Based Action Research, University of Cincinnati

**Keywords:** Community-based participatory research, citizen science, stakeholder engagement, community involvement, community participation

## Abstract

Community engagement is increasingly recognized as essential for producing impactful and ethical research outcomes. However, the field lacks a standardized, consistently used method to document community involvement, hindering transparency and accountability in the research process. To address this gap, we have developed a novel tool, the Report of Engagement in Community Research (REACH), to provide clarity on the extent, nature, and timing of community involvement in research endeavors. Unlike conventional methods that often offer vague descriptions of community participation, the REACH tool enables research teams to articulate precisely how and when community members are engaged, their specific research roles, and their level of involvement. By offering a concise, visual, structured framework, REACH empowers researchers to transparently communicate the depth and breadth of community collaboration within their projects. Using the REACH tool results in a stylized graph that describes when and what research activities community members were involved in and the extent of their collaboration. Community-academic research teams can use REACH not only to retrospectively explain community contributions to research but also to proactively plan research roles in equitable, realistic ways. By promoting transparency and accountability, the REACH tool contributes to the development of more inclusive, impactful, and ethically sound research practices. As a versatile and user-friendly tool, REACH has the potential to catalyze transformative changes in the way community-engaged research is conducted, communicated, and replicated.

Community engaged research (CEnR) has increasingly become a cornerstone of impactful and ethical research, particularly in health-related fields. The inclusion of community members in the research process is associated with positive outcomes such as healthier behaviors, policy changes, and overall health improvements (Jagosh et al., 2015; Wallerstein et al., 2020; Wallerstein & Duran, 2010) and increased potential for sustained, long term impact (Chandanabhumma et al., 2023; Jagosh et al., 2015; Kyoon-Achan et al., 2018). Within health arenas, approaches that partner with individuals with lived experience expertise are most commonly referred to as community-based participatory research (CBPR) (Israel et al., 2005; Simonds et al., 2013) or citizen science (Marks et al., 2022). We have chosen to use CEnR in this manuscript because it covers a broader scope of participatory research methods across disciplines.

Despite the recognized importance of CEnR, most academic publications do not adequately describe the role and level of engagement of community members in the research process (Medeiros et al., 2024). Research articles have word limits, and authors are understandably more likely to spend their limited real estate on the significance, approach, results, and implications of the project. The nuts and bolts of community involvement are often glossed over (e.g., “community members were involved in all aspects of the research process”), so it is difficult to assess the degree to which the research truly benefited from lived experience expertise and shared decision making. The inconsistencies in reporting can also make it challenging to ensure accountability and replicate successes. Based on a participatory research choice points framework we previously developed to conceptualize levels of engagement across the research process (Vaughn & Jacquez, 2020), we have developed the Report of Engagement in Community Research (REACH) tool to allow academic and community partners to concisely and visually describe the details of their CEnR process.

## Existing CEnR Tools

CEnR has experienced significant growth over the past two decades, leading to an increased interest in understanding and evaluating the dynamics of community-academic partnerships. Numerous tools have been developed to assess these partnerships; in the past seven years alone, at least five reviews of CEnR evaluation tools have been published, shedding light on both advancements and ongoing challenges in this field. Reviews of community-academic partnership evaluation consistently demonstrate that a substantial number of evaluation tools exist, but the field is stymied by inconsistent terminology, limited adherence to best practices in scale development and testing, and a lack of robust psychometric evidence (Boivin et al., 2018; Gordon et al., 2023; Luger et al., 2020; K. Mrklas et al., 2023; K. J. Mrklas et al., 2023). We contend that a fundamental understanding of what is actually happening when community members partner in research is essential before effective evaluation can begin.

Notably, most of the available engagement evaluation tools focus on measuring the quality of community-academic partnerships. For example, the Research Engagement Survey Tool (REST) measures adherence to eight community engagement principles (Goodman et al., 2022). The Patient Engagement in Research Scale (PEIRS) assesses meaningful engagement of patients and families as partners in research (Hamilton et al., 2018). Fewer tools are designed to report on the processes of community-academic research projects. The *Guidance for Reporting Involvement of Patients and the Public* (GRIPP2) consists of long and short form checklists for studies with patient and public involvement (PPI) as a primary or secondary focus (Staniszewska et al., 2017). The purpose of the GRIPP2 is to describe PPI-specific aims, methods, results, and discussion “to enhance the overall quality and transparency of the PPI evidence base” (p. 5). Like the GRIPP2, the Involvement Matrix (Smits et al., 2020) was developed to support PPI by assigning patients to one of five roles in each of three steps of the research process (preparation, execution, and implementation). The recently published ACCORD (ACcurate COnsensus Reporting Document) is a 35-item checklist to guide the reporting of biomedical studies using consensus methods when they are written up for publication in the biomedicine field (Gattrell et al., 2024). The ACCORD guides researchers in describing aspects of consensus-making in each section of a traditional scientific publication.

The REACH tool adds to the CEnR reporting landscape in several ways. First, it focuses tightly on communicating research methods, or the specific ways in which various community members were involved in research activities across each phase of the research process. Whereas existing measures serve projects focused on patient and public involvement, REACH can and should be completed by any team that includes community members as partners in the research, even if there is not a specific community engagement aim. Second, REACH provides a concise, visual alternative that allows a reader to quickly understand who, when, what, and to what degree community partners were involved in the research. The template is easily accessible in format and in language to individuals not trained in research, making it simple to use for community-academic research teams. REACH output provides a visual summary of community engagement that can be disseminated in journal articles, presentations, and other academic outlets as well as community settings like newsletters, emails, and funding reports.

## Development of REACH

**Figure 1.**
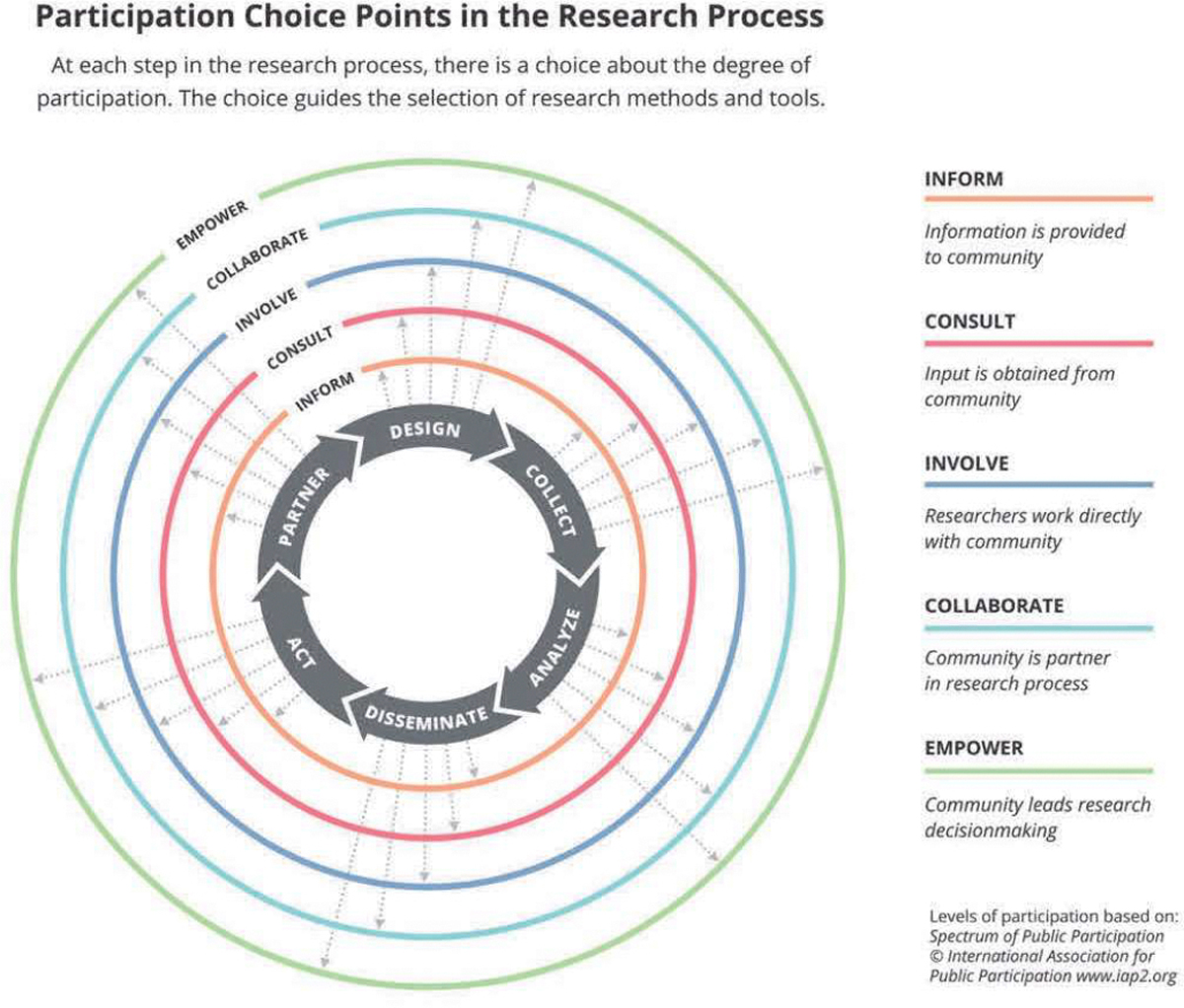
Choice Points in Community-Engaged Research (with permission from Vaughn & Jacquez, 2020)

As co-editors of the Journal of Participatory Research Methods (https://jprm.scholasticahq.com/), we conceptualized a choice points model of CEnR that frames the journal’s mission and scope (see Figure 1). The figure depicts the decisions that community-academic research teams make about the degree to which community partners are involved in each step of a cyclical research process. In our work as both editors and researchers, the choice points model has been useful in improving transparency and clarifying the ways community partners contribute to the rigor, relevance, and reach of a research study (Balazs & Morello-Frosch, 2013). As consumers of scientific literature, we are often confronted with articles that briefly indicate that community members are partners in the research without any detail about what they did or how they shared decision-making. We longed for a way to quickly understand the nuts and bolts of community engagement in articles focused on research results. We created REACH to provide a snapshot of the specific CEnR methods used in a study and the degree to which community members were involved. We worked in collaboration with a design thinking studio, Live Well Collaborative (www.livewellcollaborative.org), to translate our previously developed choice points model into a user-friendly tool that relies on widely available software (Microsoft Word and Excel) to produce a visual representation of the CEnR choices made in a project.

## Using the REACH

The REACH template and an implementation guide can be accessed from the Center for Clinical and Translational Science & Training’s website at https://www.cctst.org/reach. To begin, community-academic research teams will open the template in Microsoft Word and describe in a table who was involved and what they did in each step of the research process. For external facing dissemination, we advise teams to be as concise as possible so that the REACH output is an easily digestible visual summary of CEnR methods. For internal planning purposes, teams might choose to be more detailed to create an agreement about roles and responsibilities in the project.

Next, teams collaboratively decide which of five levels most accurately describes community involvement. Based on the International Association for Public Participation’s Spectrum of Public Participation (International Association of Public Participation (IAP2), n.d.), the five levels range from informing to empowering the community (see Table 1). Notably, the levels are not intended to be a value continuum; rather, each level of participation is a choice that teams make based on goals, resources, personnel, context, and other variables influencing how much community partners want to be involved in the research process. For example, community partners might be deeply invested in designing a study and disseminating the results but have no interest in collecting or analyzing data. REACH encourages teams to avoid the temptation to rate every research activity as highly as possible and instead to be transparent about the degree to which community partners shared decision-making and leadership in individual activities.

Teams enter the level of involvement for each research activity into an Excel file that is connected to the chart in Word. Teams can enter multiple activities at a particular stage (e.g., surveys, interviews, and focus groups in the Data Collection phase) and they will appear as connected bars on the chart. The resulting output is a bar chart that displays levels of engagement at different phases of the research process and a table with specific research activities and the community members who collaborated in them. Examples of REACH output are provided below.

### Example 1: Working with Latinx Immigrants on Social Connection Research

**Figure 2.**
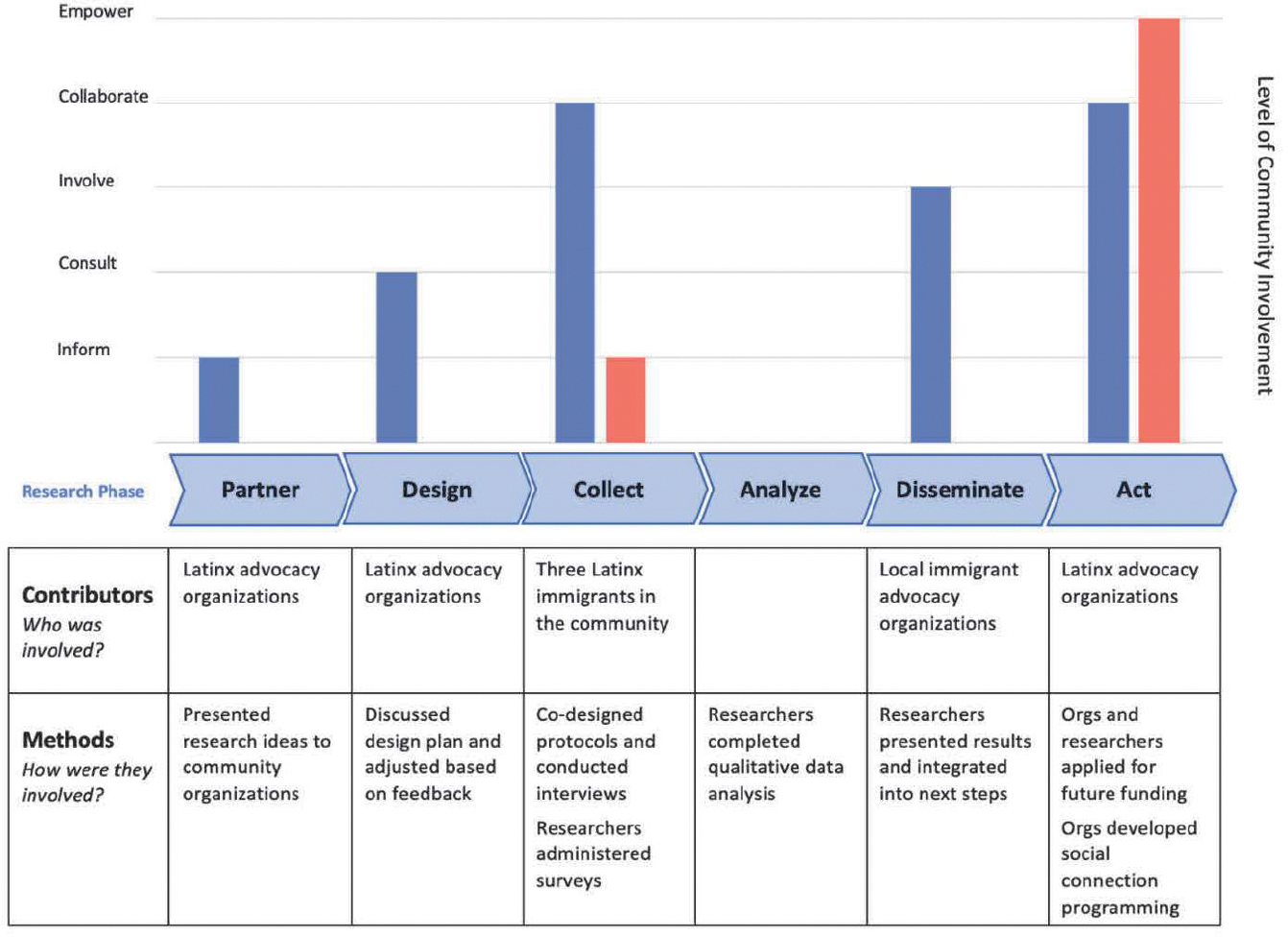
Report of community engagement in Social Connection in Immigrants project

In the spirit of transparency, our first example is one of our own studies that had more variable community engagement than most of our work with immigrant communities. After years of collaboration with community partners, we pinpointed a variety of social variables that Latinx community members report being particularly important to their wellbeing, including social acceptance, social support, and belongness (Jacquez et al., 2016, 2019; Vaughn et al., 2016). The gap in our knowledge preventing progress was an understanding of community perspectives of social connection. We conducted interviews and surveys with Latinx immigrants in Cincinnati to explore social and community foundations of health and wellness (Jacquez et al., 2024). At the outset, we met with colleagues from Latinx advocacy organizations and told them about the plans for our study. Our community colleagues provided feedback about how we should do the research, which we incorporated into the design. We hired three Latinx immigrants from the community to co-design the interview protocol and conduct all interviews. Researchers administered social connection surveys. We did not have funding for community members to be trained and conduct data analysis, so academic partners conducted this step. We shared our results with immigrant advocacy organizations throughout the area and collectively decided how to disseminate findings and next steps. In partnership with community organizations, we applied for funding to develop community interventions to improve social connection among immigrants in our region. Community organizations used the results to establish programming to improve social connection among their clients. Figure 2 depicts the REACH output from this project.

### Example 2: Working with Transgender and Gender-Diverse Youth on Decision Making Research

**Figure 3.**
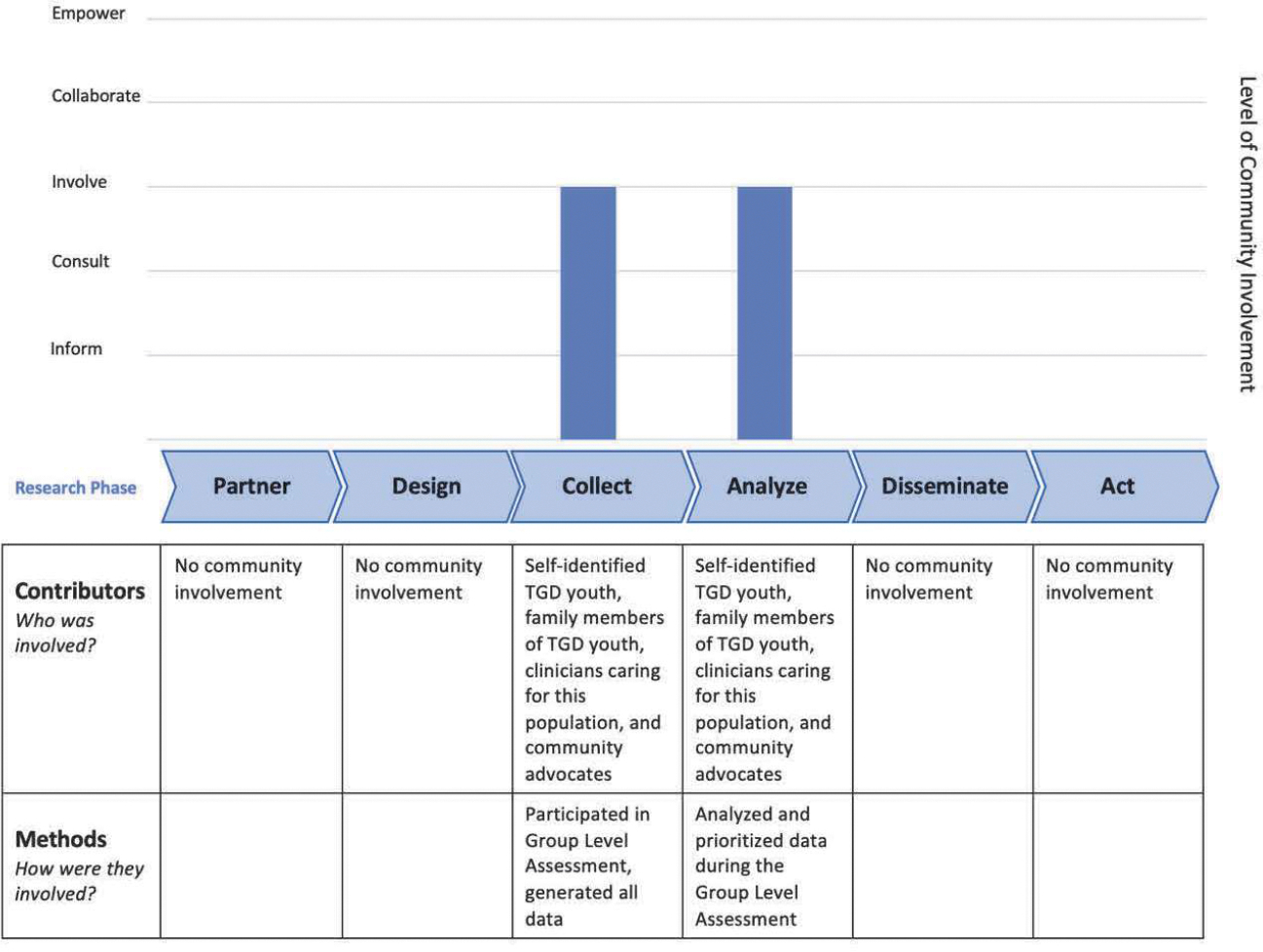
Report of community engagement in transgender decision-making research project

The second example is a pediatric patient-centered needs assessment study of transgender and gender-diverse (TGD) youth and families that used a participatory research methodology for data collection and analysis (Mazzola et al., 2023). The rationale for the study was that most decision support tools for this population did not capture the spectrum of needed decision support from the perspectives of youth and their families along with other members of the decision-making team (physicians, nurses, social workers, educators, etc.). The research team used a participatory qualitative methodology, Group Level Assessment (Vaughn, 2024; Vaughn & DeJonckheere, 2019; Vaughn & Lohmueller, 2014) to better understand the range of decisions and types of decision support needed by transgender youth and their families. In GLA, participants generated ideas and perspectives that served as the data in the project, collaboratively analyzed their data, and provided suggestions for action during the facilitated session. The decision to conduct the needs assessment, the study design, choice of method, dissemination, and action were entirely driven by the research team. The REACH output depicted in Figure 3 shows that TGD youth and families cooperated (but did not share decision-making) in collecting and analyzing data, reflecting the “involve” level in the Collect and Analyze phases of the research process. This level of community involvement was determined by the researchers and the community from the start of the project. Community partners were not mobilized to serve as full partners at every step but by being engaged in two phases of the research process, their voices were elevated, and the research was more relevant and applicable to their lives.

### Example 3: Research Led by Black Families with Autism

**Figure 4.**
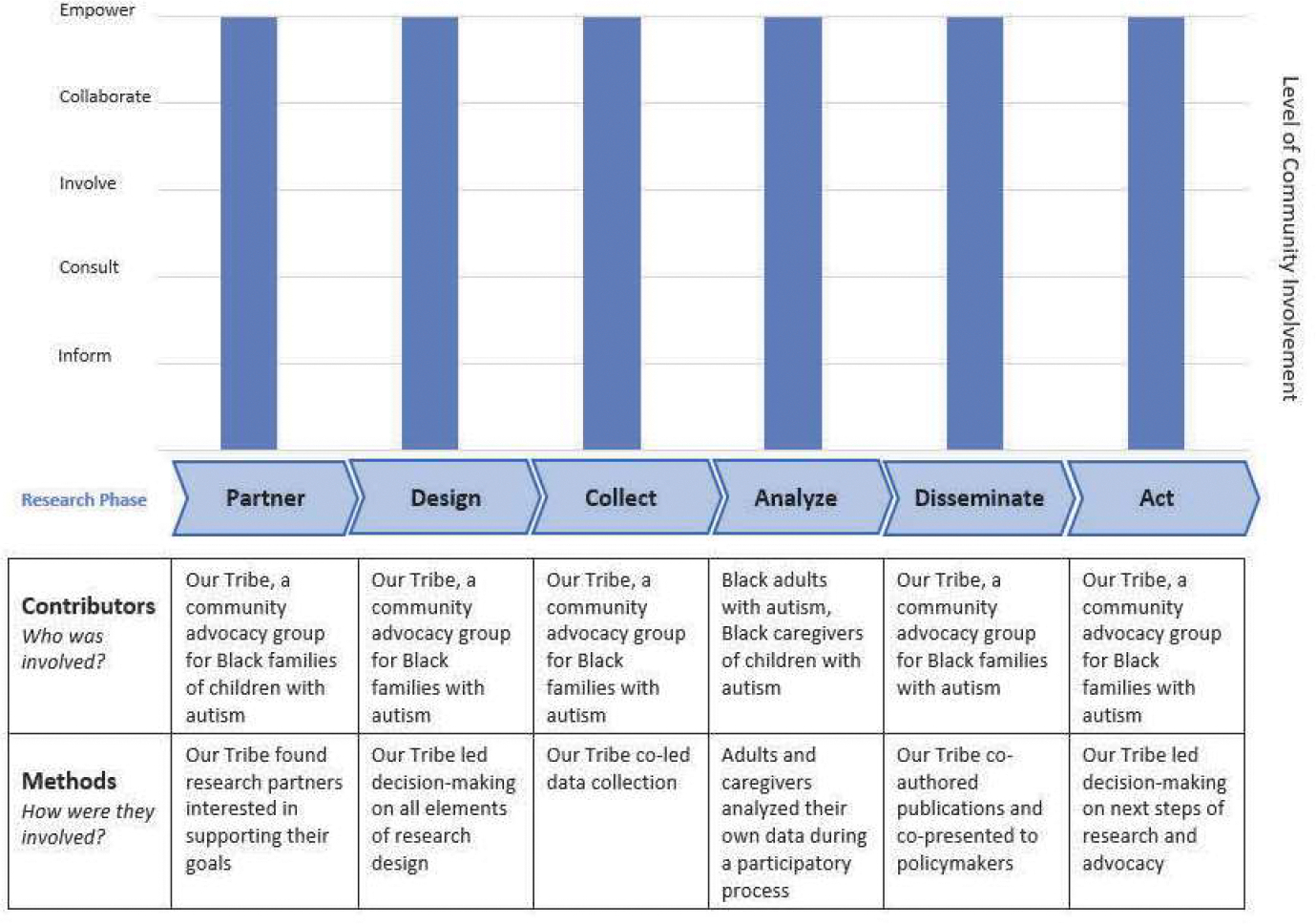
Report of community engagement in research led by Black families with autism

The third example describes a research project that was led by Our Tribe, a “grassroots initiative created by members of the Black Autism Community to uplift our families and mitigate the poor outcomes that disproportionately affect Black people diagnosed with autism” (www.ourtribecincy.com) (Castelin et al., 2022). One of Our Tribe’s goals is to conduct research centering Black families to inform policies and programs. Our Tribe found an academic partner at their local children’s hospital to provide research expertise supporting their mission. Together, they collected and analyzed data with Black families with autism and have presented their findings to local and national stakeholders. In this project, Our Tribe led with the project agenda and had the final say in all decision-making; therefore, the output reflects the “empower” level at each state of the research process (see Figure 4).

## Implications and Conclusions

The REACH tool provides a strategy to consistently report community engagement in research activities and visualize the degree of involvement of community partners. Community engagement occurs on a spectrum and is not one-size-fits-all for every community-academic partnership. Whereas some partnerships might have the capacity and interest in sharing decision-making in every step of the research process, others might prefer to partner on select activities. For instance, in example 1, Latinx advocacy organizations were invested in our long-term research partnership, but did not have the time or interest in surveys or qualitative data analysis. In Example 3, the community organization Our Tribe led every aspect of the project with researchers in more of a supporting role, exemplifying the Empowerment level at each step. In both examples, community engagement improved the quality and rigor of different aspects of the research. The purpose of REACH is not to evaluate or place a value judgement on CEnR, but to give a visual description of the choices teams made about community involvement so the reader has a comprehensive view of the process.

Although the REACH was conceptualized as a tool to retroactively describe what happened during a research project, it can also be used during the initial planning and set-up of community-engaged research projects to clarify the roles and responsibilities at each stage so that decisions are intentional and sufficient resources can be allocated accordingly. For example, in a recent community health needs assessment, a community-academic research team used REACH for planning during the initial discussions about project timeline and roles and responsibilities of community leaders, agencies, academics, and community members. This early use of REACH served as a guide through the research choice points so that community and academic partners working on the needs assessment could collaboratively discuss and find solutions to logistical barriers and facilitators to involvement before the project was underway. As the needs assessment progressed, the leadership team used REACH as a midterm check-in to adjust roles and responsibilities as needed. For instance, several of the community co-researchers realized that they didn’t have the capacity to be involved beyond the data collection stage and stepped back during the qualitative coding and analysis of interviews. The REACH tool used in this way promoted shared decision-making and fostered stronger, more meaningful collaborations. By integrating REACH into the research process, teams can better align their engagement strategies with their research objectives, leading to more inclusive, impactful, and ethically sound outcomes.

REACH has the potential to benefit CEnR by improving transparency and consistency of reporting, but the tool has limitations in scope and implementation. First, the REACH focuses on reporting community rather than researcher roles in a study. This imbalance has been criticized by some researchers as divisive because it compels equitable community-academic partners to justify the contributions of only one half of the partnership (Scholz & Bevan, 2021). We have chosen to center community roles not to justify their work but to highlight it. The nuts and bolts of community involvement is most often glossed over or not mentioned at all in traditional research articles, so we created REACH so readers can more easily comprehend how research benefited from community engagement. Second, there is a chance that REACH will not be implemented as intended and researchers will overestimate the level at which community partners shared decision-making across the phases of the research process. Although inflation of community involvement reporting is a possibility, we hope that the tool will counteract the “all or nothing” CEnR paradigm by allowing teams to accurately and concisely communicate the CEnR choices they made throughout the life of a project.

As community engagement becomes not only accepted but expected in many fields of research, consistent, transparent reporting is a critical need. REACH has the potential to serve as an easy-to-use strategy to improve transparency in reporting of community contributions in CEnR. The tool provides a concise visual depicting who was involved, what they did, and what level of shared decision-making community members had at each level of the research process. By improving transparency, we will better understand how community engagement is improving research quality, heightening the relevance of research, and broadening the impact of research on real world outcomes. Clearly communicated research involvement will allow other researchers to more easily replicate CEnR methods, which can spread best practices for improved health outcomes.

## Figures and Tables

**Table 1.** Levels of Engagement

1 INFORM	2 CONSULT	3 INVOLVE	4 COLLABORATE	5 EMPOWER
Researchers inform the community about what they are doing. Community receives information with minimal opportunity to provide perspective.	Researchers ask the community for input. Their feedback may or may not be implemented.	Researchers and the community cooperate with each other. The community is involved in the research as a partner to participate and provide feedback.	Researchers collaborate with the community and jointly make decisions with them. Partnerships are maintained through bi-directional communication.	Researchers and community collaborate; community has final decision-making power on research decisions.
